# Vertebrobasilar and Basilar Dolichoectasia Causing Audio-Vestibular Manifestations: A Case Series with a Brief Literature Review

**DOI:** 10.3390/diagnostics13101750

**Published:** 2023-05-16

**Authors:** Andrea Frosolini, Francesco Fantin, Valeria Caragli, Leonardo Franz, Salvatore Fermo, Ingrid Inches, Andrea Lovato, Elisabetta Genovese, Gino Marioni, Cosimo de Filippis

**Affiliations:** 1 Phoniatris and Audiology Unit, Department of Neuroscience DNS, University of Padova, 31100 Treviso, Italyleonardo.franz@unipd.it (L.F.);; 2 Maxillofacial Surgery Unit, Department of Medical Biotechnologies, University of Siena, 53100 Siena, Italy; 3 Audiology Unit, Department of Diagnostic, Clinical and Public Health Medicine, University of Modena and Reggio Emilia, 41100 Modena, Italy; 4 Artificial Intelligence in Medicine and Innovation in Clinical Research and Methodology (PhD Program), Department of Clinical and Experimental Sciences, University of Brescia, 25100 Brescia, Italy; 5 Neuroradiology Unit, Treviso Hospital, 31100 Treviso, Italy; 6 Otorhinolaryngology Unit, Department of Surgical Specialties, Vicenza Civil Hospital, 36100 Vicenza, Italy

**Keywords:** vertebrobasilar, basilar, artery, dolichoectasia, hearing loss, tinnitus, vertigo, audio-vestibular

## Abstract

Audio-vestibular symptoms can arise from vertebrobasilar dolichoectasia (VBD) and basilar dolichoectasia (BD). Given the dearth of available information, herein we reported our experience with different audio-vestibular disorders (AVDs) observed in a case series of VBD patients. Furthermore, a literature review analyzed the possible relationships between epidemiological, clinical, and neuroradiological findings and audiological prognosis. The electronic archive of our audiological tertiary referral center was screened. All identified patients had a diagnosis of VBD/BD according to Smoker’s criteria and a comprehensive audiological evaluation. PubMed and Scopus databases were searched for inherent papers published from 1 January 2000 to 1 March 2023. Three subjects were found; all of them had high blood pressure, and only the patient with high-grade VBD showed progressive sensorineural hearing loss (SNHL). Seven original studies were retrieved from the literature, overall including 90 cases. AVDs were more common in males and present in late adulthood (mean age 65 years, range 37–71), with symptoms including progressive and sudden SNHL, tinnitus, and vertigo. Diagnosis was made using different audiological and vestibular tests and cerebral MRI. Management was hearing aid fitting and long-term follow-up, with only one case of microvascular decompression surgery. The mechanism by which VBD and BD can cause AVD is debated, with the main hypothesis being VIII cranial nerve compression and vascular impairment. Our reported cases suggested the possibility of central auditory dysfunction of retro-cochlear origin due to VBD, followed by rapidly progressing SNHL and/or unnoticed sudden SNHL. More research is needed to better understand this audiological entity and achieve an evidence-based effective treatment.

## 1. Introduction

Elongation, widening, and tortuosity of the vessels in the posterior brain circulation are defined as vertebrobasilar dolichoectasia (VBD) and basilar dolichoectasia (BD) depending on the involved anatomical structures. With a prevalence ranging from 0.06% to 5.8% and a reported male predominance, VBD is due to a deterioration of the tunica structure given by multifactorial etiologies affecting both basilar and vertebral arteries, while BD is limited to the basilar artery [1]. The conditions may remain silent or induce a wide range of symptoms which can be slowly progressive or acute and life-threatening. Regarding the latter, an increased cumulative risk of stroke of 15.9% at 10 years from diagnosis has been reported [2]. On the other hand, considering non-life-threatening symptoms, nearly 40% of patients with VBD can have disorders related to the VIII cranial nerve (CN), which is the most commonly involved one among the twelve CNs [1]. Consequently, VBD and BD may induce vertigo, tinnitus, and hearing loss [3,4]. These symptoms have been predominantly related to two different mechanisms: (i) thrombotic–ischemic vascular mechanisms, as the cochlea and vestibular system have a terminal vascularization and receive a limited blood supply without collateral circulation; (ii) a compressive mechanism, as the elongated and tortuous arteries can directly displace the surrounding tissues, including CNs and brainstem [5]. The local increment in pro-inflammatory cytokines may play a relevant role in the vascular pathogenetic mechanism [6]. A recent article reported an association between vertebrobasilar ectasia, neutrophil-to-lymphocyte ratio (NLR), a circulating inflammatory marker, and hearing loss [7]. In addition, as nerves traversing the cerebellopontine angle (CPA) cistern can be compressed by VBD, patients may report progressive VIII CN deficits [8].

Therefore, the possibility of VBD as a cause of audio-vestibular dysfunction must be considered, along with neurovascular conflict between the anterior inferior cerebellar artery (AICA) and the vestibulocochlear nerve and CPA masses [3,4,9]. Cerebral contrast-enhanced magnetic resonance imaging (MRI) and angio-TC are the milestones of VBD and BD diagnosis. A commonly accepted way to classify VBD and BD severity is currently lacking, although Smoker’s criteria, based on radiological findings, including laterality, bifurcation height, and basilar artery diameter, are often employed [1].

At the same time, specific recommendations for clinical management of VBD and BD still need to be developed, and a long-term follow-up is commonly employed as a precautionary approach even for asymptomatic cases [1].

Given the dearth of available information, the main aim of this investigation was to report our experience with the spectrum of different audio-vestibular disorders observed in a case series of VBD diagnosed during a 7-year period. Furthermore, a literature review was performed in order to analyze the possible relationships between epidemiological, clinical, and neuroradiological findings and audiological prognosis.

## 2. Materials and Methods

Data were examined in accordance with the Helsinki Declaration, the Italian privacy and sensitive data laws, and the in-house regulations of our hospital. All patients gave their written consent for clinical case publication. The electronic archive (Talete) of our tertiary referral center for audio-vestibular disorders (Phoniatrics and Audiology Unit, University of Padova, Treviso, Italy) was consulted. The time framework evaluated ranged from 2015 to 2022. Inclusion criteria were the following: (i) diagnosis of vertebrobasilar dolichoectasia according to Smoker’s criteria [10]; (ii) audio-vestibular symptoms; and (iii) available neuroimaging. Data about three patients were retrieved; all of them had undergone complete physical otolaryngologic evaluation, audiological test evaluation, and neuroradiological study. Case reports were done following the CARE checklist.

### 2.1. Audiological Tests

Audiometry was performed with Madsen Astera by GN Otometrics (Taastrup, Denmark), in accordance with European (IEC 60645-I) and ISO (389-1) standards, in a sound-attenuating room, as previously reported [11]. Hearing thresholds were tested with and without hearing devices, in the best aided condition, at frequencies ranging from 250 to 8000 Hz. Pure tone average (PTA2, threshold levels at 0.5, 1, 2, and 4 kHz) was then calculated. For speech audiometry, articulation gain curves were obtained using disyllabic, phonetically balanced words from an Italian wordlist for adults [12]. The speech reception threshold (SRT) decibel level at which 50% of words could be repeated by the patients was considered.

Distorted product otoacoustic emission (DPOAE) was registered with the ILO 292 system (Version 6, Otodynamics Ltd., Hatfield, UK), recorded at 1000, 1501, 2002, 3003, 4004, 6006, and 7996 Hz (f2). Impedance was studied using GSI TympStar Version 2 (Eden Praire, MN, USA).

Auditory brainstem potentials (ABRs) were recorded from scalp electrodes (vertex to mastoid ipsilateral to the stimulated ear) for 2000 trials of alternating polarity clicks presented monaurally using a TDH-50 transducer earphone at a maximum intensity of 125 dB p.e. SPL (corresponding to 90 dB nHL, referring to the psycho-acoustical threshold of normally hearing subjects). The filter settings of the amplifier were set between 5 and 4000 Hz.

Spontaneous/positional nystagmus was registered with VisualEyes Goggles and VNG software v7, Interacoustic (Middelfart, Denmark). The procedure was followed by a bi-thermic caloric instrument test using an Otometrics ICS AirCal (Taastrup, Denmark) with air at 50 °C and 24 °C, 60″ of stimulation per side, and 70″ of recording (including 10″’ of visual fixation).

### 2.2. Neuroradiological Evaluation

MRI protocol included T1-W and 3D T2-W sequences of the skull base and the posterior fossa. Subsequently, contrast-enhanced or time-of-flight (TOF) magnetic resonance angiography (MRA) was performed. The inclusion of a sequence covering the whole head, a T1-W, T2-W, or FLAIR sequence, needed to be considered for the evaluation of intracranial space-occupying lesions or vascular abnormalities.

Multi-detector CT angiography (CTA) or CT venography (CTV) of the head and neck region was performed with detector configurations that covered the whole head with 16 cm coverage as 320 × 0.5 mm or 256 × 0.625 mm collimations, as previously described [13]. A temporal resolution of up to 20 frames/s was achieved from a continuous volume acquisition.

### 2.3. Literature Review

PubMed and Scopus electronic databases were searched for papers on vertebrobasilar dolichoectasia published in the last 22 years (from 1 January 2000 to 1 March 2023). The search strings were as follows: “basilar dolichoectasia tinnitus” OR “basilar dolichoectasia hearing” OR “basilar dolichoectasia vertigo” OR “vertebrobasilar dolichoectasia tinnitus” OR “vertebrobasilar dolichoectasia hearing” OR “vertebrobasilar dolichoectasia vertigo” for PubMed; (basilar AND dolichoectasia AND tinnitus) OR (basilar AND dolichoectasia AND hearing) OR (basilar AND dolichoectasia AND vertigo) OR (vertebrobasilar AND dolichoectasia AND tinnitus) OR (vertebrobasilar AND dolichoectasia AND hearing) OR (vertebrobasilar AND dolichoectasia AND vertigo) for Scopus. The “Related articles” option on the PubMed homepage was also considered. Titles and abstracts of papers available in the English language were examined. The identified full texts were screened for original data, and the related references were retrieved and checked manually for other relevant studies. Studies were included when the following general criteria were met: (i) articles were original reports; (ii) studies included only clinically confirmed cases of audiological symptoms in patients with vertebrobasilar dolichoectasia; (iii) investigation reported detailed information about diagnostic workout and/or treatment results; (iv) study design was not editorial, letter to the editor, or review; (v) the report was written in the English language. Included studies were analyzed to extract all available data and ensure eligibility for all patients. Descriptive data of epidemiology, etiology, symptoms, diagnostic procedures, treatments, outcomes, and follow-up were considered.

## 3. Results

### 3.1. Case Series (Table 1)

#### 3.1.1. Case 1

A 66-year-old male patient complaining of left tinnitus in the previous 7 months was referred to our Unit. The patient’s medical history was relevant for high blood pressure (HBP) under medical treatment. Otoscopy examination was bilaterally normal. The pure-tone audiometry showed bilateral mild sensorineural hearing loss (PTA 20 dB); speech audiometry was bilaterally within normal limits (SRT 30 dB). Tympanograms were normal. Acoustic reflexes were partially present. DPOAEs were present bilaterally. ABR showed increased latencies of wave V on the left ear (7.01 ms left ear; 5.74 ms right one). The Tinnitus Handicap Index score (THI) was 28/100.

**Figure 1:**
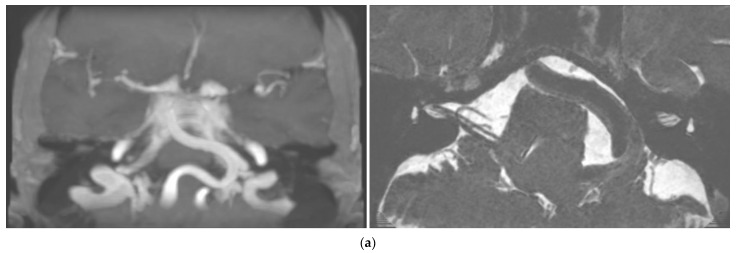

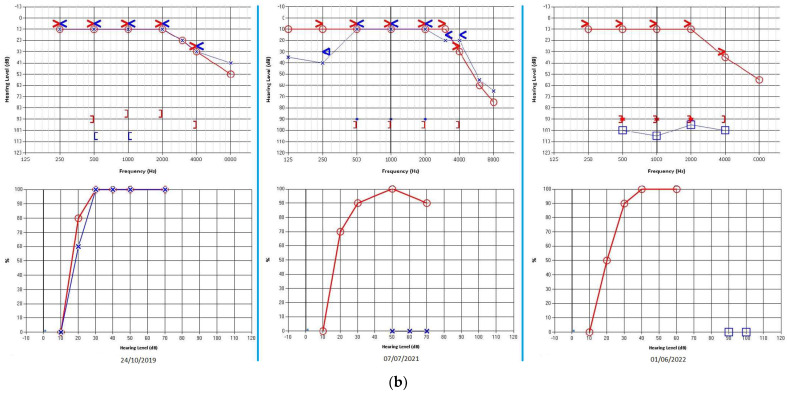
Case 1: (**a**) Cerebral MRI images of Case 1 showing vertebrobasilar dolichoectasia of grade 7/7 (Smoker’s classification) determining dislocation of the VIII CN. (**b**) Progressive hearing decrement of the patient at three different controls during the 31−month follow-up period. Right ear: red graphs; left ear: blue graphs; bone conduction: arrowheads; air conduction (right): circles; air conduction (left): crosses/squares (masked); square brackets: acoustic reflex threshold.

At 6-month follow-up, the patient reported dizziness and severe hearing loss in the left ear. The patient also reported poor blood pressure control. PTA was unchanged but SRT was absent on the left side, and ABR waves on the left side were not detectable. DPOAEs were bilaterally present. Upon vestibular examination, spontaneous nystagmus was absent, and left vestibular hyporeflexia was found in the caloric test. THI was 12/100. Brain MRI showed normal inner ear structures but revealed the presence of vertebrobasilar dolichoectasia (Smoker classification: 7/7) and postero-lateral dislocation of the left branches of VII and VIII CNs (Figure 1a). According to clinical manifestations and exams, a left retro-cochlear hearing loss due to compression/stretching of the vestibular acoustic nerve due to VBD was diagnosed. Due to the location of the vascular abnormalities and the non-life-threatening symptoms, there was no indication for surgery. A close follow-up of blood pressure values and cardiovascular risk factors was planned, along with a cerebral CT angiography and a repetition of cerebral MRI at 12 months. The patient was fitted with a bilateral contralateral routing of signals (CROS) hearing aid system and reported subjective benefit in his daily activities.

One-year follow-up radiological images showed unchanged conditions; the patient did not report any different symptoms. Nonetheless, pure tone audiometry revealed aggravation of hearing loss on the left side (PTA 90 dB) with unilateral absent DPOAE (Figure 1b).

#### 3.1.2. Case 2

**Figure 2:**
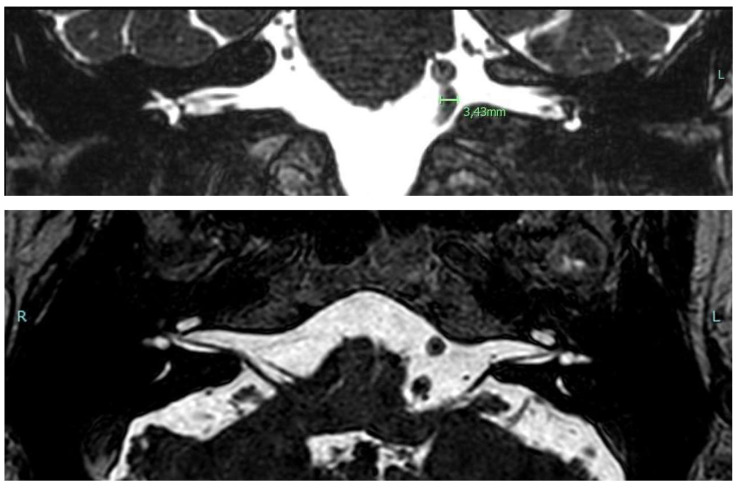
Cerebral MRI images in Case 2 showing grade 5/7 (Smoker’s classification) basilar dolichoectasia.

A 68-year-old male patient complaining of bilateral hearing loss and ear fullness (worse on the left side) was referred to our Unit to undergo a course of Eustachian tube insufflations. The patient also suffered from bilateral tinnitus in the previous 10 years and recurrent episodes of benign paroxysmal positional vertigo (BPPV), as previously diagnosed and treated in our clinic, according to international diagnostic criteria [14,15]. HBP under medical treatment was reported. Otoscopy was normal. Pure tone audiometry showed a bilateral mild to moderate asymmetric sensorineural hearing loss (PTA 31.25 dB on the right ear; 51.25 dB on the left one). Speech recognition tests revealed reduced SRT on the left side (37 dB on the right ear vs. 67 dB on the left one). Tympanograms were normal with the presence of contralateral acoustic reflexes bilaterally. An on–off effect (diphasic impedance change) on the left side was found. ABR revealed a slight delay of the fifth wave on the left side (5.80 ms on the right ear vs. 5.89 ms on the left one). Brain MRI (Figure 2) showed white matter lesions in the centrum semiovale, a normal representation of the acoustic-facial package on both sides, and a dolichobasilar artery (Smoker classification 5/7).

The patient was fitted with bilateral hearing aids with subjective and objective benefit at three-year follow-up control, without significant progression of the hearing loss.

#### 3.1.3. Case 3

**Figure 3:**
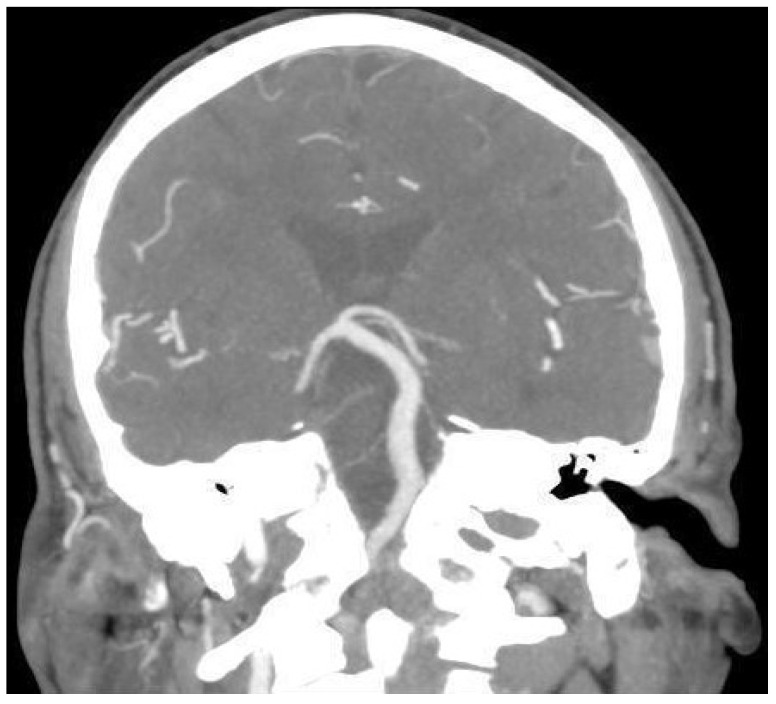
Cerebral CT angiography image of Case 3, showing 2/7 (Smoker’s classification) basilar dolichoectasia.

An 80-year-old male patient was referred to our Unit complaining of bilateral subjective hearing loss, dizziness, and ear fullness to perform a course of Eustachian tube insufflations. Past history was relevant for renal carcinoma surgically treated three years before and HBP under medical therapy. Otolaryngological examination was within normal limits. Pure tone audiometry showed bilateral sensorineural hearing loss (PTA 45 dB on the right ear; PTA 45 dB on the left one). Speech audiometry was consistent with tonal audiometry (SRT 60 dB on the right ear; SRT 55 dB on the left one). ABR showed a slight delay of the fifth wave on the left side (5.56 ms on the right ear vs. 5.64 ms on the left one). Tympanograms were normal. Acoustic reflexes were bilaterally present. Videonystagmography (VNG) showed no signs of vestibular anomalies, as reported in Table 1. An angio-CT scan was performed (Figure 3), showing moderate cortical atrophy with widespread chronic vascular encephalopathy for multi-infarction and marked hypodensity of the periventricular white matter. Moreover, the imaging showed the presence of a dolichobasilar artery (Smoker classification: 2/7).

Hearing aids were prescribed but the patient refused for personal reasons. A cardio-vascular visit found scarce control of blood pressure; its normalization brought to dizziness regression. Follow-up controls showed no significant progression of hearing loss.

**Table 1:** Main demographic and clinical parameters of the three new cases reported.

Patient	Age (Years) and Sex	Main Comorbidities	Symptoms	DPOAE	ABR	PTA	SRT	Vestibular Tests	Smoker’s Classification	Treatment	Follow-Up	Audiological Prognosis
1	66 M	HBP	Tinnitus, dizziness, SNHL (LS)	Altered	Altered	Altered	Altered	Altered	7/7	CROS HA	2 years	Progression to deafness
2	68 M	HBP	Tinnitus, SNHL (LS), PPV	Altered	Normal	Altered	Altered	Altered	5/7	Bilateral HA	3 years	No progression
3	80 M	HBP	SNHL symmetrical, dizziness	Altered	Normal	Altered	Normal	Normal	2/7	None	3 years	No progression

List of abbreviations: ABR: auditory brainstem response, CROS: contralateral routing of signals, DPOAE: distorted product otoacoustic response, HA: hearing aid, HBP: high blood pressure, LS: left side, M: male, PPV: paroxysmal positional vertigo, PTA: pure tone audiometry, SNHL: sensorineural hearing loss, SRT: speech recognition threshold.

### 3.2. Literature Review

**Figure 4:**
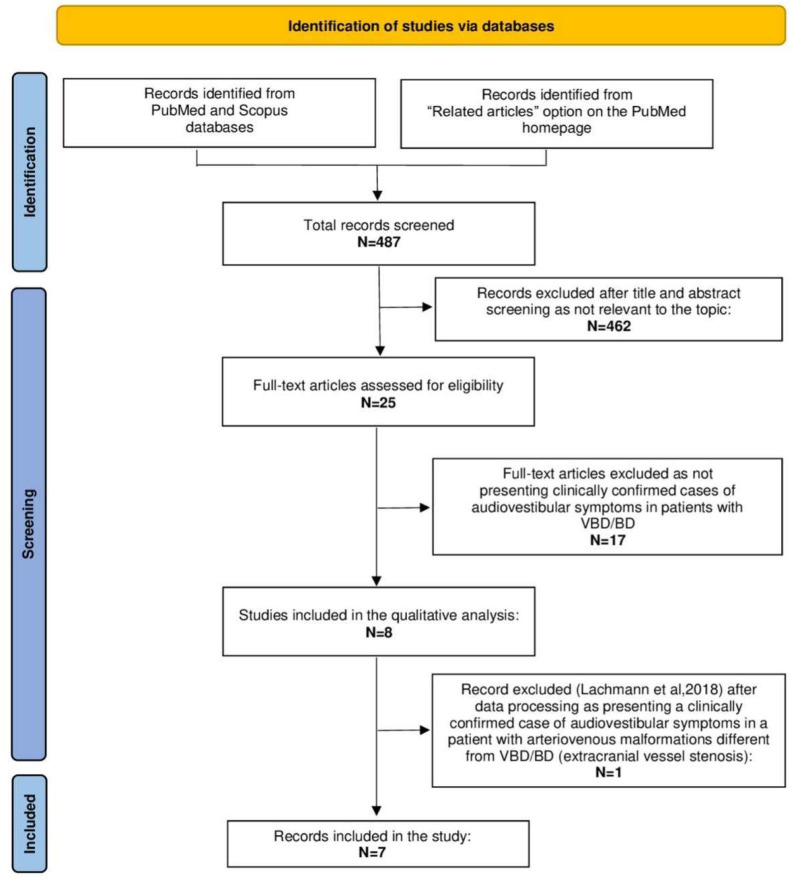
Preferred Reporting Items for Systematic Reviews and Meta-Analyses (PRISMA) diagram summarizing the review process from research to inclusion. **List of abbreviations**: BD: basilar dolichoectasia, N: number, VBD: vertebrobasilar dolichoectasia.

Following our research methods, 487 manuscripts were retrieved from the PubMed and Scopus databases from January 2000 to March 2022 (see also Figure 4). Seven articles [4,6,9,16,17,18,19] were included in the present study, according to inclusion/exclusion criteria. Table 2 summarizes the results of this review.

A total of 90 patients (48 males, 32 females) have been analyzed; the mean age was 65 years (range 37–71). Predominant cochleovestibular symptoms included hearing loss, sudden hearing loss, tinnitus, and vertigo. In two cases, hemifacial spasm and facial pain were reported. The majority of patients (71%) had a medical history of hypertension, syncopal events, and cardiovascular disorders.

Considered patients generally developed mild to moderate hearing loss, without significant alterations of speech recognition [4,6,9,16]. When ABRs were performed, augmented latencies of I–V and III waves were recorded [9,19]. Vestibular assessment tools included Dix–Hallpike test, caloric test, VNG, head impulse test (HIT), and head shaking Test (HST) [4,9,16,17,18]. MRI was performed for all cases. VBD [8,9,16,17,18] was more frequently reported than BD [6] with 85 cases vs. 5 cases, respectively. Smoker’s classification was applied in only one study [4] to quantify dolichoectasia; the other studies used different methods to report the alterations in the posterior circulation vessels (see Table 2). As for the treatment, conservative management was chosen in the majority of cases, while only one subject underwent surgical microvascular decompression [8]. Patients were followed from 6 months [4] to 5 years [4]; five out of seven included manuscripts did not report the follow-up period.

## 4. Discussion

VBD is a vascular condition first described by the international literature in the early 20th century; the term VBD is often used to encompass both vertebral and basilar artery involvement. It is important to differentiate between VBD and BD as they can have different clinical presentations, radiological features, and potential complications [20]. Additionally, the diagnostic criteria and definitions of VBD and BD have not been standardized, leading to potential inconsistencies in the available evidence [21]. VBD and BD were usually diagnosed incidentally during imaging studies, such as MRI or CT scans [22]. The first reports of audio-vestibular symptoms due to VBD and BD date back to the second half of the 20th century [3]. Nonetheless, to date, some uncertainty remains concerning the epidemiology, pathophysiology, classification, and treatment of audio-vestibular disorders in VBD and BD.

As mentioned before, the exact mechanism by which VBD and BD can cause audio-vestibular symptoms is still debated. One of the main hypotheses is VIII CN compression, as postulated by Passero and Rossi [5], which may cause signs/symptoms with a mechanism similar to that of CPA lesions [23]. Vestibular schwannoma and other less common CPA lesions (e.g., meningiomas, epidermoid tumors, and arachnoid cysts) generate a compression of the cochlear nerve and then lead to a decrease in neural activity and progressive degeneration of nerve fibers over time [24].

Moreover, vascular mechanisms leading to blood flow alterations in the vertebrobasilar system, resulting in a reduction in blood supply to the cochlea, have been advocated in CPA pathologies [7]. In the case of VBD and BD, there is no general consensus on which side could suffer more from such flow impairments. In fact, some research groups [7] claimed that the augmented BA’s diameter could cause homolateral blood flow decrement, resulting in cochleovestibular symptoms on the same side, whereas others hypothesized flow alterations contralateral to the side of the vascular anomaly [25] or even bilateral [3].

Decreased cerebral perfusion of posterior circulation territory characterized by delayed time-to-perfusion in dynamic, susceptibility-weighted, contrast-enhanced perfusion cerebral MRI was shown in a study on 77 patients with VBD, resulting in an augmented risk of posterior ischemic stroke [18].

Few studies have focused on the management of neurological symptoms related to VBD and BD [26]. Concerning trigeminal neuralgia due to BD/VBD-based neurovascular conflict, promising results have been shown by microvascular decompression [27,28], although overall evidence is still limited.

On the other hand, in a prophylactic setting, preliminary good results have been shown by the administration of novel oral anticoagulants to prevent stroke [18]. Regarding the treatment of audio-vestibular symptoms, very limited reports are available, accounting only for small, retrospective series.

In the studies included in this review, the treatment of audio-vestibular disorders was always conservative, except for one case, in which surgical microvascular decompression (MVD) was indicated, due to the coexistence of a hemifacial spasm (HFS) on the same side of the audio-vestibular symptoms [29]. In this case, the left HFS was relieved immediately after surgery, while there was a worsening of the hearing loss [4].

In all the considered studies, BD or VBD diagnosis was based on cerebral MRI. However, only one provided a classification of BD/VBD severity according to Smoker’s criteria [4].

**Figure 5:**
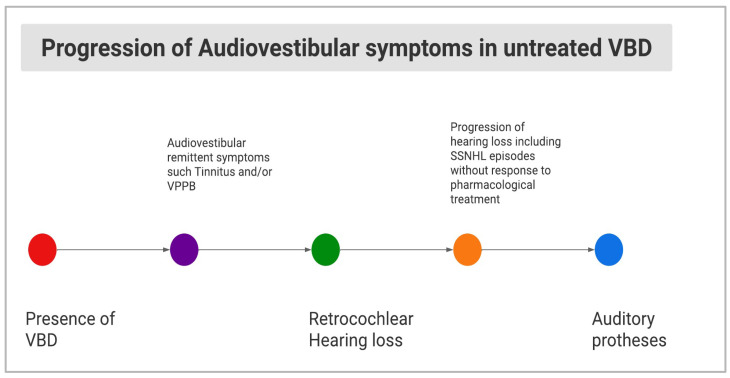
Diagram showing hypothesis of the natural history of audio-vestibular signs/symptoms in untreated VBD.

In the considered articles, the clinical evaluation of audio-vestibular signs/symptoms was based on different tests, including pure tone and speech audiometry, ABR, vHIT, and VNG (see Table 2). Interestingly, no cases of hearing loss progression similar to Case 1 of our series were reported in the available literature (see Figure 1b and Figure 5).

In the present study, we reported the case series of three patients with different audio-vestibular symptoms (tinnitus, aural fullness, subjective hearing loss, and dizziness); all these patients developed various degrees of sensorineural hearing loss on the same side of their VBD or BD. Given the advanced age of the patients, we should not neglect the contribution of presbyacusis to the hearing decrement, especially concerning Case 3 for whom age-related hearing loss has been considered as a differential diagnosis. In such cases, we stress the need for an accurate hearing diagnosis and a consequent prompt treatment to avoid late-insufficient hearing aid fitting [30]. Regarding vestibular involvement, we found vestibular nerve hypofunction in the caloric test in Case 1, in accordance with previous findings of vestibular loss in patients with VBD. On the other hand, Case 2 had suffered from PPV for years, before VBD diagnosis was defined. It is not possible to establish a clear causal relationship between BPPV and VBD for this patient; however, some pathophysiological considerations can be applied to this case. Although to the best of our knowledge, there are no reports of BPPV attributed to VBD in the literature, a significantly higher rate of vertebral artery (VA) tortuosity has been reported in a prospective study on 104 patients with BPPV [31]. According to Zhang et al. [31], VA tortuosity may lead to decreased blood flow, collapse and occlusion of the blood vessels, and atherosclerosis, and therefore the distal perfusion of the inner ear may be compromised, ultimately exacerbating BPPV [31]. Moreover, vestibular paroxysmia has been reported in VBD patients due to direct compression of the vestibular nerve and needs to be considered in differential diagnosis [4,17]. Further studies are necessary to explore the etiopathogenic role of VBD in vertigo’s genesis and differential diagnosis.

Regarding possible risk factors, all three patients of our series showed HBP as a comorbidity. Interestingly, our literature review showed that HBP was present in more than half of reported cases with VBD/BD and audio-vestibular disorders. Regarding other possible epidemiological associations resulting from the literature, a slight male prevalence (109 vs. 81 cases) and a late adult mean age (mean: 65 years; range: 27–81 years) were found.

The size of our series did not allow us to investigate any statistical association; however, some findings deserve to be mentioned: (i) only the case with the highest Smoker’s classification score (7/7) showed progression of hearing loss (see Table 1); (ii) the patient who had hearing loss progression was also the youngest. The latter finding seems to be consistent with what has been previously reported in the literature, with a younger age at presentation being related to the clinical progression of hearing loss [29].

The natural history of our Case 1 may present some interesting insights into the possible pathophysiology of audiological symptoms in VBD. In the first months of clinical presentation, Case 1 developed a retro-cochlear left hearing loss as shown by normal PTA and DPOAE and the absence of SRT and ABR waves (see Table 1 and Figure 1b). This clinical–instrumental scenario resembles the integrity of the peripheral hearing system and the cochlea and alterations at the central hearing pathway levels. Nonetheless, during the consequent follow-up, the clinical picture progressed through complete left-side deafness, as shown by severe hearing loss in PTA (>90 dB) and the absence of left-side DPOE.

It is well known that certain types of hearing disorders may be due to brain dysfunction, referring as central auditory dysfunction (CAD). Models of CAD, apart from pediatric central auditory processing disorders, include acquired auditory neuropathy of the VIII CN due to retro-cochlear lesions, in its most common form due to vestibular schwannoma [23,32]. Our Case 1 could have shown a CAD of retro-cochlear origin due to VBD followed by rapidly progressing SNHL and/or unnoticed SSNHL (Figure 5). Therefore, this intriguing case suggested a double pathophysiological hypothesis, in which both compressive and vascular mechanisms related to VBD prompted, in sequence, the audiological dysfunction.

For all our audiological patients, we preferred a conservative treatment based on hearing aid fitting and long-term follow-up. In fact, in the absence of sufficient evidence supporting any kind of active treatment of VBD (except for the case of life-threatening complications), a potential surgical approach would have led to a potentially unfavorable risk–benefit balance, in particular regarding hearing outcome, as previously reported [33]. Nonetheless, an intriguing approach to consider could be the therapeutic combination of MVD and cochlear implantation (CI), in analogy with the recent growing indication of CI after vestibular schwannoma resection [34].

Overall, in the present study, we reported our experience with the management of patients with audio-vestibular symptoms and VBD. The main strengths of our study are the application of a complete audiological evaluation and the long-term follow-up; the main limitations are related to the retrospective monocentric study design.

## 5. Conclusions

Audio-vestibular manifestations of VBD and BD have been reported for many decades, but the epidemiological and clinical features, etiopathogenesis, and best management strategy are still unclear. Herein, we reported a clinical scenario in which the natural history of audio-vestibular symptoms of VBD/BD could be explained by both compressive and vascular mechanisms.

Based on the small amount of available evidence, a long-term follow-up seems to be a useful approach, along with continued monitoring of the possible evolution of signs/symptoms in collaboration with neurologists and neuroradiologists and the early detection of possible complications. However, further studies (also including large retrospective multicentric series and randomized control trials) are mandatory to better understand this audiological entity with the objective of identifying an evidence-based effective therapeutic approach.

## Figures and Tables

**Table 2:** Summary of the results retrieved from our literature review.

First Author, Year	N of Patients, Age, Gender	Comorbidities	Symptoms	ABR	PTA	SRT	Vestibular Test	Radiological Findings (MRI)	Treatment	Follow-Up Time
Kang, 2022 [6]	5 cases (3M, 2F) 52.1 (27–81 y)	HBP, CVD	HL, vertigo	-	-	-	-	Bad > 4.5 mm	-	-
Rodríguez, 2022 [16]	1 case M 74 y	HBP	HL, vertigo, syncopal events	-	RS: moderate (borderline severe) SNHL	Speech discrimination in the RS of 100% at 100 dB	HIT, skew test, pursuit, VNG, Dix–Hallpike, hyperextension, roll test	VBD (RS)	Oral steroids	-
Huh, 2020 [4]	4 cases (2M, 2F) 61.5 (37–71 y)	-	HL, tinnitus, vertigo, hemifacial spasm	LS: > I–V (ID: 0.4 ms)	Case 1 LS: mild HL Case 3 RS: moderate HL Case 4: LS: down-sloping HL	-	VNG, HST, HIT, caloric test	VBD (BA bifurcation grade 2 * BA lateral position grade 1–3 *)	MVD, Ginkgo biloba, Tianeptine, Oxcarbazepine	28 m
Gilbow, 2018 [9]	1 case F 63 y	-	HL, tinnitus, vertigo	-	LS: down-sloping mild-to-severe SNHL	LS: <discrimination, rollover	Head thrust test	VBD (LS), IAC herniation, VII and VIII CN displacement	-	-
Han, 2018 [17]	1 case F 66 y	HBP	Vertigo, tinnitus, facial pain and spasm	-	-	-	Dix–Hallpike test	VBD (RS), V and VIII CN compression	B1 vitamin, Methylcobalamin, Carbamazepine	6 m
Peng, 2018 [18]	77 cases (51M, 26F) 63 y	HBP, CVD	Vertigo	-	-	-	Caloric test, Dix–Hallpike test, roll test	VBD	-	-
Titlic, 2007 [19]	1 case M 73 y	HBP	Tinnitus, vertigo	LS: >I–III wave, <amplitude	-	-	-	VBD (LS)	Betahistine, Ticlopidine	-

List of abbreviations: ABR: auditory brainstem response, BA: basilar artery, BAD: basilar artery dolichoectasia, Bad: basilar artery diameter (mm), BAL, basilar artery length, BL, bending length, CN: cranial nerve, CVD: cardiovascular disease, DPOAE: distorted product otoacoustic response, HIT: head impulse test, HBP: high blood pressure, HL: hearing loss, IAC: inner auditory canal, ID: interaural difference, LS: left side, m: months, MDV: microvascular decompression, NH: normal hearing, PTA: pure tone audiometry, RS: right side, SNHL: sensorineural hearing loss, SRT: speech recognition threshold, TI, tortuosity index, VA: vertebral artery, VAD: vertebral artery dolichoectasia, VNG: videonystagmography, y: years. * Smoker’s criteria.

## Data Availability

In respect of privacy, the data obtained are available upon reasonable request.
